# Editors’ Note

**DOI:** 10.5195/ijt.2018.6261

**Published:** 2018-08-03

**Authors:** ELLEN R. COHN, JANA CASON

## ISSUE OVERVIEW

Where is the *International Journal of Telerehabilitation* (IJT) positioned among journals in the telemedicine and telehealth space? IJT is an online, open (i.e., subscription free) journal that focuses on telerehabilitation -- a segment of telehealth and telemedicine that encompasses many rehabilitation associated disciplines. Because successful telerehabilitation often depends upon interprofessional collaborations, much of the work published within IJT is by at least two authors from different disciplines. Authors in the current issue include persons with collective expertise in the disciplines of architecture, occupational therapy, medicine, physical therapy, and/or software engineering.

An examination of the subject matter published in IJT since its inception in 2008 provides insight into the evolution of telerehabilitation. Readers will note that several current issue papers describe telerehabilitation services delivered in the home environment, with one investigating in-home modifications. Two articles studied the provision of telehealth to persons post-stroke. The therapeutic use of virtual environments is a relatively newly prominent theme.

We posit that multiple factors influence emerging research themes: reimbursement for services rendered, regulations, and most importantly in the case of research articles, trends in research funding. International submissions to the IJT have also shifted, sometimes between continents. The international geographic trends are interesting to follow.

In the current issue, readers will note cautions related to safety and patient selection. Moreover, since some telerehabilitation protocols are best hybrid (i.e., variously incorporating both in-person and online assessments and interventions), IJT welcomes articles that include research conducted in authentic, hybrid conditions. Appropriate patient selection for telerehabilitation services is broached in this issue, and remains a challenge that requires future research.

Sincerely,

Ellen R. Cohn, PhD, CCC-SLP, ASHA-F, IJT Editor

Jana Cason, DHSc, OTR/L, FAOTA, Senior Associate Editor

